# *Propionibacterium acnes*-associated chronic hypertrophic pachymeningitis followed by refractory otitis media: a case report

**DOI:** 10.1186/s12883-020-1600-3

**Published:** 2020-01-09

**Authors:** Eiichiro Amano, Keisuke Uchida, Tasuku Ishihara, Shinichi Otsu, Akira Machida, Yoshinobu Eishi

**Affiliations:** 1 Department of Neurology, Tsuchiura Kyodo General Hospital, 4-1-1 Otsuno, Tsuchiura-shi, Ibaraki 300-0028 Japan; 2 Division of Surgical Pathology, Tokyo Medical and Dental University Hospital, 1-5-45 Yushima, Bunkyo, Tokyo, 113-8519 Japan; 3 Department of Human Pathology, Graduate School and Faculty of Medicine, Tokyo Medical and Dental University, 1-5-45 Yushima, Bunkyo, Tokyo, 113-8519 Japan

**Keywords:** *Propionibacterium acnes*, Otitis media, Pachymeningitis, Mastoiditis, Dura mater

## Abstract

**Background:**

Hypertrophic pachymeningitis (HP) is a rare disorder that involves localized or diffuse thickening of the dura mater. HP is associated with various inflammatory, infectious, and malignant diseases, such as rheumatic arthritis, sarcoidosis, anti-neutrophil cytoplasmic antibody-associated vasculitis, IgG4-related disorders, syphilis, tuberculosis, bacterial and fungal infections, cancer, and idiopathic diseases, when evaluation fails to reveal a cause. Among them, chronic infection with *Propionibacterium acnes* is a rare etiology of HP, and its pathology remains unclear.

**Case presentation:**

An 80-year-old man having refractory otitis media with effusion of the right ear presented with progressive right-sided headache and nausea. Post-contrast brain magnetic resonance imaging revealed right mastoiditis and remarkable thickening of the dura mater and enhancement of pia mater extending from the right middle cranial fossa to the temporal lobe.

HP secondary to middle ear infection was suspected, and a biopsy of the right mastoid was performed. An anaerobic culture of the biopsied right mastoid showed the growth of *P. acnes*, and histopathological examination using *P. acnes*-specific monoclonal antibody (PAB antibody) revealed the infiltration of inflammatory cells with *P. acnes*. Moreover, using PAB antibody, *P. acnes* was detected in the biopsy specimen of the thickening dura mater. No granulomas were identified in either specimen. HP was resolved with long-term administration of antibiotics and steroids.

**Conclusion:**

This is the first documentation of pathologically demonstrated chronic HP associated with *P. acnes* infection followed by refractory otitis media. This report showed that chronic latent *P. acnes* infection induces chronic inflammation.

## Background

Hypertrophic pachymeningitis (HP) is a rare disorder that involves localized or diffuse thickening of the dura mater [1]. Most patients present with chronic headaches with or without other neurological manifestations such as cranial nerve palsies, cerebellar ataxia, seizures, myelopathy, and visual disturbances [2]. HP is associated with various inflammatory, infectious, and malignant diseases, such as rheumatic arthritis, sarcoidosis, ANCA-associated vasculitis, IgG4-related disorders, syphilis, tuberculosis, bacterial and fungal infections, cancer, and idiopathic’ diseases, when evaluation fails to reveal a cause [3]. Among them, chronic infection with *Propionibacterium acnes* (*P. acnes*) is a rare etiology of HP [4].

*P. acnes* is a slow-growing, non-spore forming anaerobic gram-positive bacillus that ubiquitously resides in human skin and hair follicles [5]. Intriguingly, *P. acnes* can invade and persist in epithelial cells and circulating macrophages, thus inducing chronic inflammation [5]; it is considered the most implicated etiological agent for sarcoidosis in Japan because it has been isolated by bacterial culture from systemic sarcoidosis lesions at high ratios [6].

Regarding the association of *P. acnes* with the intracranial infections, it is one of the important pathogens responsible for postoperative meningitis and subdural empyema [7–9]. However, several cases of chronic meningitis and HP due to *P. acnes* have been reported which were not associated with neurosurgical procedures or trauma [10–12]. Moreover, in some cases, both antibiotics and the concomitant use of steroids alleviated the disease course [13, 14].

Here we report the first case of HP due to pathologically proven chronic *P. acnes* infection, which was resolved by long-term administration of both antibiotics and steroids.

## Case presentation

An 80-year-old man presented with deafness and otorrhea of right ear and was diagnosed as having otitis media with effusion (OME). Myringotomy, the insertion of a ventilation tube, and administration of multiple oral antibiotics (cefditoren pivoxil (CDTR-PI) 300 mg/day for 2 weeks, sitafloxacin (STFX) 100 mg/day for 19 days, and clarithromycin (CAM) 400 mg/day for 2 weeks) were only partially effective; his OME had been recurrent and refractory to these treatments. Although myeloperoxidase and proteinase 3 anti-neutrophil cytoplasmic antibodies (MPO-ANCA, PR3-ANCA) were negative in the blood examination, otitis media with ANCA-associated vasculitis was suspected, and prednisolone (30 mg/day) was orally administered [15] without antibiotics. However, because the improvement in deafness and otorrhea was transient and insufficient, prednisolone administration was tapered off for a period of 4 weeks.

Six months later, a chronic right-sided headache emerged and gradually worsened. Eight months after the onset of OME, he suffered from nausea and severe headache and was admitted to our hospital.

**Fig. 1:**
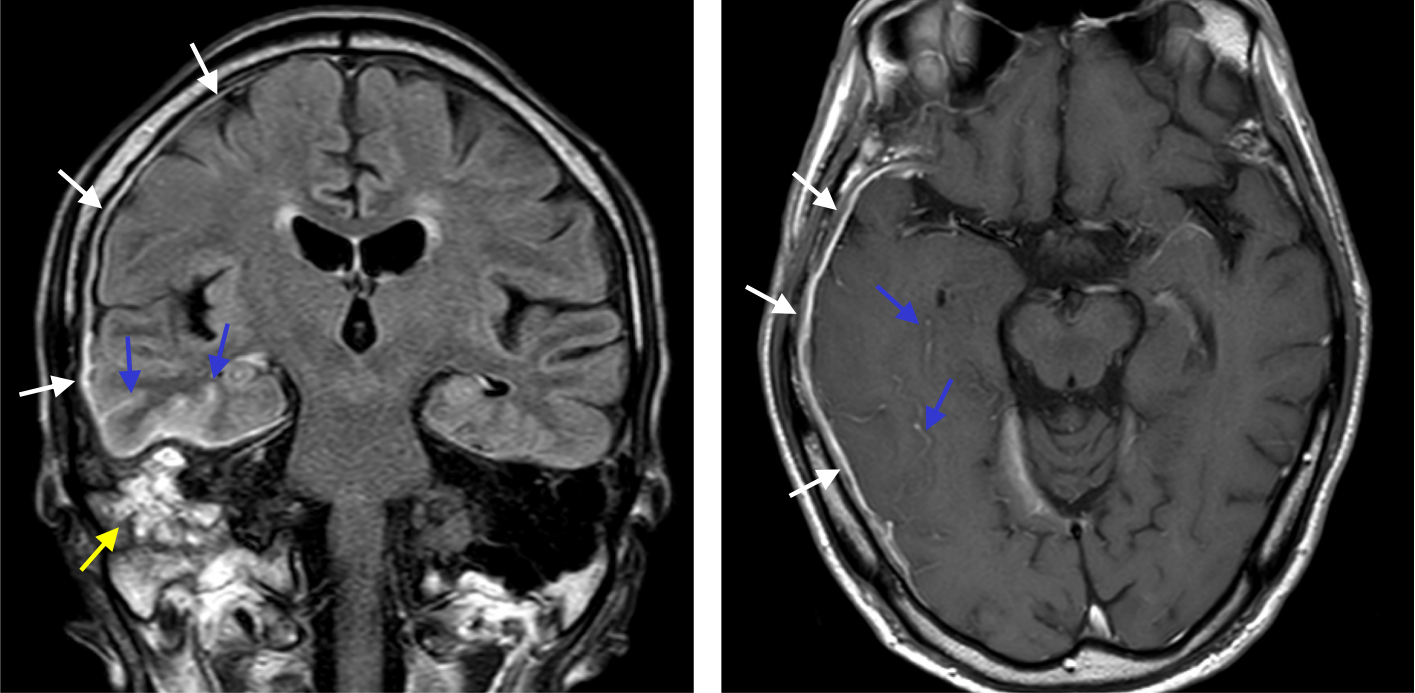
Hypertrophic pachymeningitis. Coronal section (left) of post-contrast fluid-attenuated inversion recovery (FLAIR) on MRI, and axial section (right) of post-contrast T1 on MRI. Gadolinium enhancement of the dura mater in the right temporal lobe and the middle cranial fossa (white arrows), and enhancement of the pia mater (blue arrows) Right mastoiditis was also evident (yellow arrows)

Post-contrast MRI revealed right mastoiditis, remarkable thickening of the dura mater, and enhanced pia mater extending from the right middle cranial fossa to the temporal lobe (Fig. 1). Cerebrospinal fluid (CSF) examination revealed an elevated cell count (31/mm^3^, mononuclear cells 30/mm^3^) and total protein levels (91 mg/dl). Repeated CSF cultures were negative, and the results of the polymerase chain reaction analyses performed on the CSF for *Mycobacterium tuberculosis*, Epstein–Barr virus, Cytomegalovirus, and Herpes simplex virus were also negative. CSF cytology was normal, and no oligoclonal band was detected. Neither cryptococcal antigen testing nor Indian ink staining of CSF was positive.

Both MPO-ANCA and PR3-ANCA were evaluated again in the serum; however, the results were negative. An additional blood examination showed normal angiotensin-converting enzyme, lysozyme, and immunoglobulin G4 levels. Anti-CCP antibodies, rheumatoid factor, and anti-nuclear antibodies were not detected. Serology test results for syphilis, human immunodeficiency virus, human T-lymphotropic virus type 1, candida, and aspergillus were all negative. *Mycobacterium tuberculosis* was not present as per the results of the interferon-gamma release assays. βD-glucan was not detected in the blood. C reactive protein levels were slightly elevated (1.19 mg/dl).

**Fig. 2:**
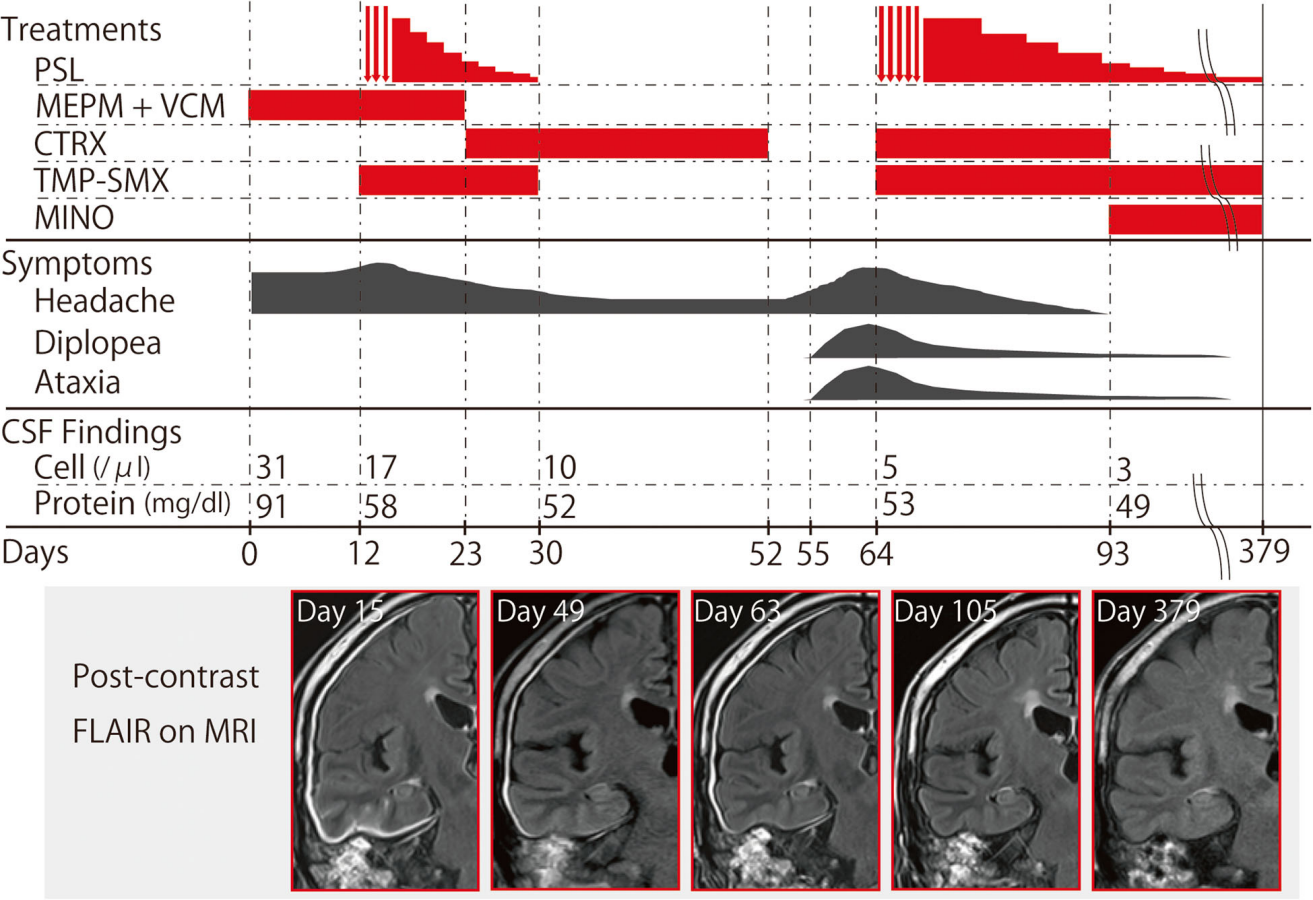
An overview of treatments, symptoms, CSF findings, and radiological findings on MRI. PSL: predonisolone (red arrows in treatments indicate high-dose IVMP), MEPM: meropenem, VCM: vancomycin, TMP-SMX: trimethoprim-sulfamethoxazole, MINO: minocycline, CTRX: ceftriaxone. Post-contrast FLAIR on MRI revealed that gadolinium enhancement of the dura mater, pia mater, and mastoid was diminished after the long-term administration of PSL, TMP-SMX, and MINO

**Fig. 3:**
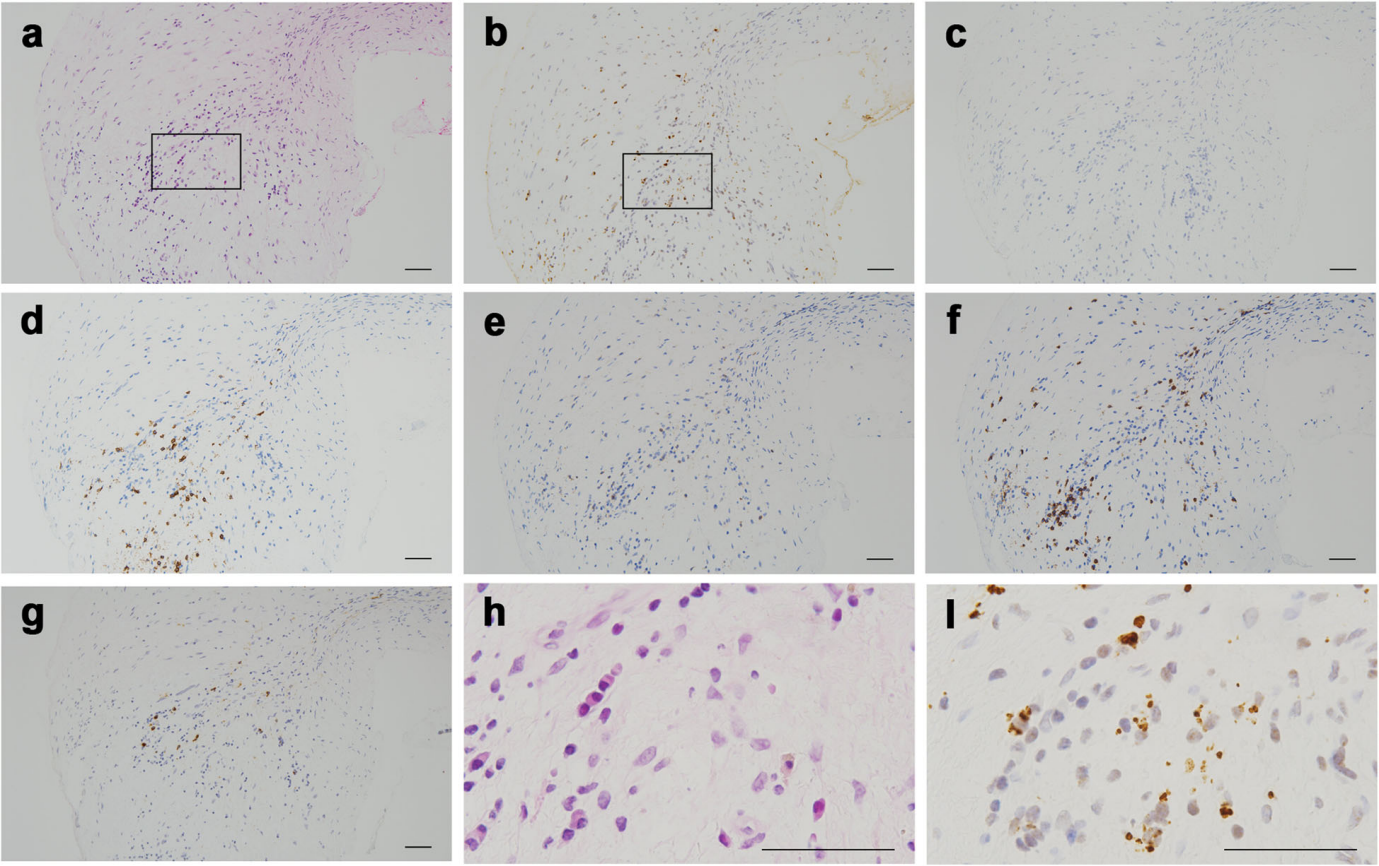
A biopsied mastoid tissue. **a** HE. **b** *Propionibacterium acnes* stain. **c** Mycobacteria stain. **d** CD20 stain. **e** CD4 stain. **f** CD8 stain. **g** IgG4 stain. **h**, **i** Enlarged views of the HE and Propionibacterium stains, which are enclosed in the squares in (**a**) and (**b**), respectively. Bars: 50 μm

HP secondary to bacterial middle ear infection was suspected, and meropenem (MEPM) (6 g/day) and vancomycin (VCM) (2 g/day) were administered. Although the CSF examination showed decreased cell counts (19/mm^3^) and protein levels (58 mg/dl) on day 13, the patient’s headache gradually worsened, and a post-contrast MRI showed no improvement (Fig. 2). A biopsy of the right mastoid was performed, and *P. acnes* was detected using *P. acnes*-specific monoclonal antibody (PAB antibody); culture results for *P. acnes* were also positive (Fig. 3, discussed below).

To control the post-infectious intracranial inflammation, high-dose intravenous methylprednisolone (IVMP) (1000 mg/day) was administered for 5 days followed by oral prednisolone (45 mg/day), and his headache was remarkably alleviated. Because *P. acnes* is sensitive to ceftriaxone (CTRX), MEPM and VCM were switched to CTRX (4 g/day). Although the patient received predonisolone (PSL), trimethoprim-sulfamethoxazole (TMP-SMX) was also prescribed for prophylaxis against pneumocystis pneumonia [16]. PSL was stopped after 4 weeks, and CTRX was administered for 7 weeks. The post-contrast MRI showed improved dural thickening, and the patient’s headache disappeared.

**Fig. 4:**
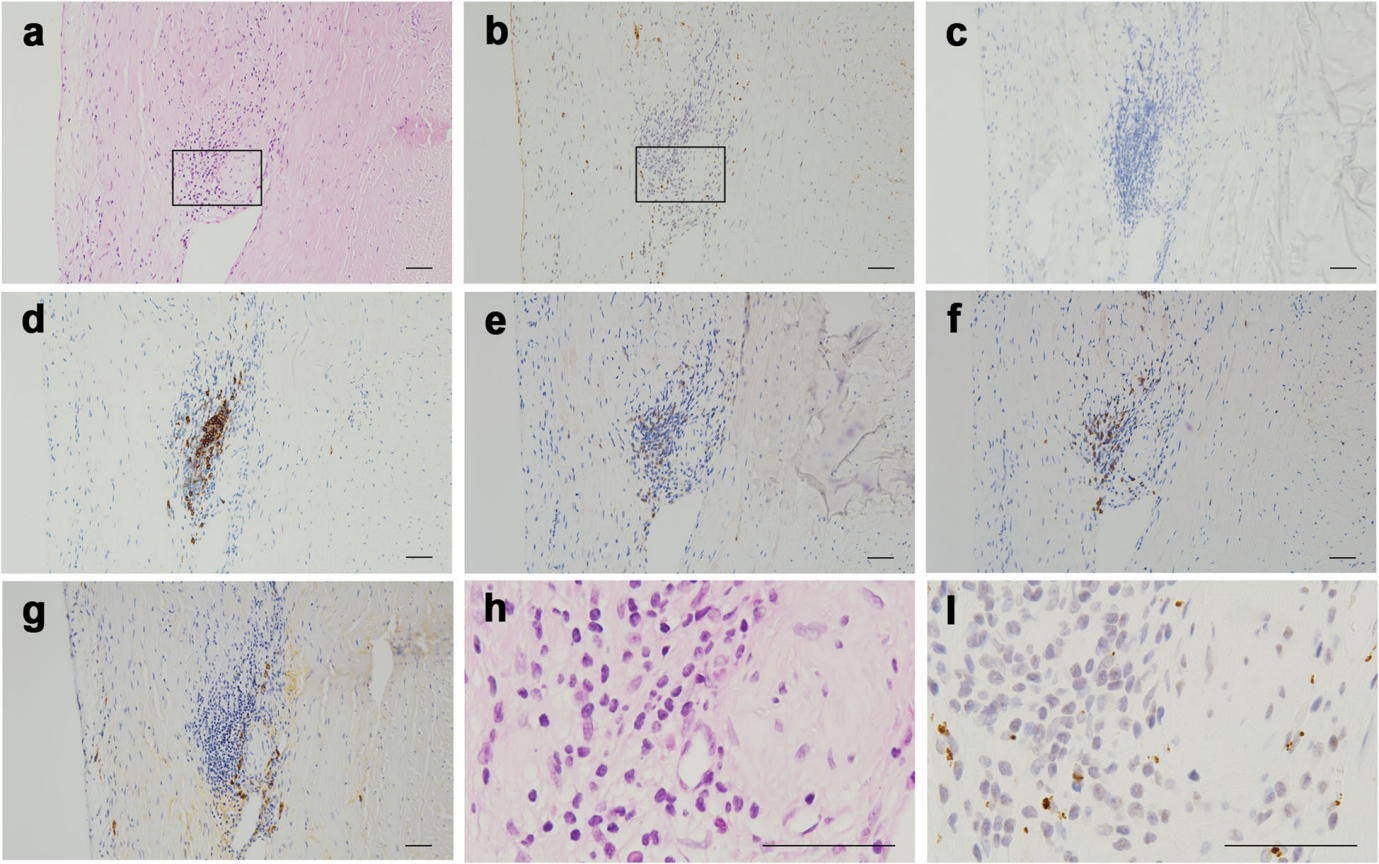
A biopsied tissue from the dura mater. **a** HE. **b** *Propionibacterium acnes* stain. **c** Mycobacteria stain. **d** CD20 stain. **e** CD4 stain. **f** CD8 stain. **g** IgG4 stain. **h**, **i** Enlarged views of the HE and *Propionibacterium acnes* stains, which are enclosed in the squares in (**a**) and (**b**), respectively. Bars: 50 μm

However, HP relapsed 4 weeks after the discontinuation of PSL and 1 week after the discontinuation of CTRX (Fig. 2). The patient experienced severe headache, gait disturbance, and diplopia. Neurological examination revealed right abducent paralysis and ataxia. We performed a biopsy of the dura mater; *P. acnes* was detected using PAB antibody; however, it was not detected in the culture of the dura mater (Fig. 4, discussed below). After we performed a biopsy of the dura mater, high-dose IVMP (1000 mg/day) was administered for 5 days, followed by oral prednisolone (45 mg/day). CTRX (4 g/day) was simultaneously administered for 4 weeks and then switched to oral minocycline (MINO). TMP-SMX had been concomitantly administered since high-dose IVMP was initiated. After we restarted the treatment, his symptoms gradually disappeared. PSL was tapered but had been continued with both MINO and TMP-SMX for 1 year. Even after finishing these three medications, the patient has remained free from relapses for more than 1 year.

### Pathological findings

No granulomas were identified, but inflammatory cell infiltration with concomitant post-inflammatory fibrosis was evident in both samples based on HE staining (Fig. 3a and h, Fig. 4a and h). No organisms were identified by HE and PAS staining.

Based on reports and the literature, formalin-fixed and paraffin-embedded tissues of the right mastoid and dura mater were tested using immunohistochemical methods with PAB antibody [6] and mycobacterial species-specific monoclonal antibody [17], which were generated according to the protocol previously reported; appropriate controls were also used. Many small round bodies were detected by immunohistochemistry with PAB antibody in the inflammatory macrophages of both tissues (Fig. 3b and i, Fig. 4b and i). Immunoreactivity for mycobacterial species was not identified within either of the tissues (Fig. 3c, Fig. 4c). Immunoreactivity for *P. acnes* was more evident in the right mastoid than in the dura mater. CD4-, CD8-, or CD20-positive cells had infiltrated around the PAB-positive small round bodies (Fig. 3d–f, Fig. 4d–f). There were more CD8-positive cells in both tissues than CD4-positive cells. IgG4 cells were scattered, and the IgG4/IgG cell ratio was less than 40% in these inflammatory lesions (Fig. 3h, Fig. 4h).

## Discussion and conclusions

*P. acnes* is a part of commensal microbiota and is generally present in human skin, hair follicles, and mucous membranes [5, 18]. It is well known for its involvement in the pathogenesis of acne vulgaris [19]; moreover, its role in various postoperative infections such as endocarditis, endophthalmitis, and central nervous system (CNS) infections has been reported [5]. In addition, *P. acnes* can invade and persist in epithelial cells and circulating macrophages in its intracellular latent form [5], which results in chronic inflammation such as sarcoidosis [6, 20, 21].

CNS infections caused by *P. acnes* are generally associated with previous neurosurgical procedures, such as craniotomy and cerebrospinal shunt placement [7–9]. Brain abscess, subdural or epidural empyema, and shunt meningitis are common *P. acnes*-related CNS infections that are successfully treated by antibiotics, sometimes in combination with surgical interventions such as drainage and debridement [7–9]. By contrast, *P. acnes* causes meningitis without neurosurgical procedures in both immunocompromised [22] and immunocompetent patients [10–14]. In *P. acnes* infection cases without trauma or surgical procedures, the paths by which *P. acnes* invades the CNS putatively include (1) hematogenous routes via the diploic veins from the hair follicles or sebaceous glands of the scalp or (2) invasion from adjacent inflammation such as sinusitis, orbititis, and otitis media [4]. Anaerobic bacteria, including *P. acnes,* are important pathogens in otitis media [23] and are sometimes found in the cultures of chronic secretory otitis media [24]. In this case, chronic inflammation in OME created an anaerobic condition, which is advantageous for *P. acnes* colonization and biofilm production; consequently, *P. acnes* subsequently invaded the adjacent intracranial meninges.

Because *P. acnes* has often been considered a common contaminant of blood and fluid cultures, we analyzed the biopsied mastoid by immunohistochemistry with PAB antibody to exclude the possibility that the *P. acnes* in the fluid cultures from the biopsied right mastoid was a consequence of false-positive contamination. In addition, although the CSF culture did not test positive for *P. acnes*, it was successfully detected by immunohistochemistry with PAB antibody within the biopsied dura mater and mastoid.

Yang et al. reported a case of *P. acnes*-associated neurosarcoidosis identified by immunohistochemical analysis of the brain tissue using PAB antibody [25]. Contrarily, our case differs from sarcoidosis because pathological analyses did not reveal the presence of non-caseating granulomas. Furthermore, there was greater CD8-positive lymphocyte infiltration than CD4-positive lymphocyte infiltration in the specimens, which is distinct from sarcoidosis because CD4-positive lymphocytes are dominant in sarcoidosis [21]. The mechanism by which CD8-dominant inflammation was triggered was unclear; however, *P. acnes* antigens in the infected tissues putatively induced the cytotoxic immune response and exaggerated the inflammatory reaction, which cannot be controlled without the administration of steroids.

Although *P. acnes*-associated CNS infection can be treated with antibiotics in most cases, French et al. reported a rare case of *P. acnes*-associated meningitis responsive to steroids [14]. Conversely, Yamashita et al. reported a case of chronic meningitis that was successfully treated by steroids without antibiotics; this patient subsequently died of acute meningitis caused by *P. acnes* [13]. Although his case did not prove the presence of *P. acnes* in CSF during the treatment of chronic meningitis and it was unclear whether *P. acnes* actually caused the chronic meningitis, we should be aware that steroid administration against chronic *P. acnes* infection without antibiotics might result in a fatal outcome. Except for sarcoidosis, which has been reported to be caused by a hypersensitive Th1 immune response to *P. acnes* and can thus be treated by steroids, chronic *P. acnes* infection should be carefully observed when steroids are administered.

Notably, otitis media sometimes accompanies HP in anti-neutrophil cytoplasmic antibody (ANCA)-associated vasculitis without systemic manifestations such as pulmonary and renal involvement [1, 26]. It was difficult to discern between ANCA-associated vasculitis and chronic *P. acnes*-associated inflammation without either a histopathological examination or blood testing of ANCA in this patient.

We should be aware that this report has limitations of being based on a single case: in order to determine the effectiveness of the treatment for *P. acnes*-associated chronic HP, future study which uncover the clinical course of multiple patients with the same disease will be needed.

In conclusion, we report the first known case of *P. acnes*-associated chronic HP followed by refractory otitis media. The patient was successfully treated by long-term administration of antibiotics and steroids. Further study is necessary to reveal how *P. acnes* induce chronic latent infection and inflammation in the brain and establish treatment strategies to control the chronic inflammation.

## Data Availability

The datasets used and/or analyzed during the current study are available from the corresponding author on reasonable request.

## Abbreviations

ANCA: Anti-neutrophil cytoplasmic antibody
CAM: Clarithromycin
CDTR-PI: Cefditoren pivoxil
CNS: Central nervous system
CSF: Cerebrospinal fluid
CTRX: Ceftriaxone
HP: Hypertrophic pachymeningitis
IVMP: Intravenous methylprednisolone
MEPM: Meropenem
MINO: Minocycline
MPO: Myeloperoxidase
OME: Otitis media with effusion
PAB antibody: *P. acnes*-specific monoclonal antibody
PR3: Proteinase 3
PSL: Prednisolone
STFX: Sitafloxacin
TMP-SMX: Trimethoprim-sulfamethoxazole
VCM: Vancomycin
